# Low-Cost Eye-Tracking Fixation Analysis for Driver Monitoring Systems Using Kalman Filtering and OPTICS Clustering

**DOI:** 10.3390/s25227028

**Published:** 2025-11-17

**Authors:** Jonas Brandstetter, Eva-Maria Knoch, Frank Gauterin

**Affiliations:** 1 Faculty of Mechanical Engineering, Institute for Vehicle Systems Engineering, Karlsruhe Institute of Technology (KIT), 76131 Karlsruhe, Germany; eva-maria.knoch@kit.edu (E.-M.K.); frank.gauterin@kit.edu (F.G.); 2 Department of Complete Vehicle Data Driven Testing, Analysis and Vehicle Functions, Porsche Engineering Services GmbH, 74321 Bietigheim-Bissingen, Germany

**Keywords:** driver monitoring, eye tracking, fixation analysis, Kalman filter, OPTICS clustering

## Abstract

Driver monitoring systems benefit from fixation-related eye-tracking features, yet dedicated eye-tracking hardware is costly and difficult to integrate at scale. This study presents a practical software pipeline that extracts fixation-related features from conventional RGB video. Facial and pupil landmarks obtained with MediaPipe are denoised using a Kalman filter, fixation centers are identified with the OPTICS algorithm within a sliding window, and an affine normalization compensates for head motion and camera geometry. Fixation segments are derived from smoothed velocity profiles based on a moving average. Experiments with laptop camera recordings show that the combined Kalman and OPTICS pipeline reduces landmark jitter and yields more stable fixation centroids, while the affine normalization further improves referential pupil stability. The pipeline operates with minimal computational overhead and can be implemented as a software update in existing driver monitoring or advanced driver assistance systems. This work is a proof of concept that demonstrates feasibility in a low-cost RGB setting with a limited evaluation scope. Remaining challenges include sensitivity to lighting conditions and head motion that future work may address through near-infrared sensing, adaptive calibration, and broader validation across subjects, environments, and cameras. The extracted features are relevant for future studies on cognitive load and attention, although cognitive state inference is not validated here.

## 1. Introduction

Driver condition assessment is increasingly critical in modern vehicles. Human error accounts for 94% of all traffic accidents [1], underscoring the need for real-time systems capable of assessing driver condition and mitigating risks. The growing adoption of DMS is driven by regulatory mandates and technological advancements. For instance, the European Union’s 2019 regulation [2] requires new vehicles to incorporate systems that monitor driver availability, fatigue, and attention. Additionally, the demand for personalized and comfort-enhancing features, such as adaptive Heating, Ventilation, and Air Conditioning (HVAC) systems and individualized ADAS settings, has further fueled interest in DMS technologies [3]. At its core, driver condition assessment involves observing the driver and deriving conclusions about their vital, emotional, cognitive, and activity states based on biofeedback and vital signals. Vital data primarily include physiological measurements such as heart rate, body temperature, or respiratory rate. Biofeedback data extends to additional physiological parameters, such as muscle tension, skin conductivity, and fixation parameters from eye tracking. The presented approach is used to determine such fixation characteristics.

This paper does not propose a new tracking or clustering algorithm. It provides a proof of concept that reliable fixation-related features can be extracted from ordinary RGB video using a careful integration of existing algorithms. The work focuses on feasibility and implementation aspects, demonstrating that such a software only approach can stabilize gaze tracking without specialized sensors. Broader validation across subjects, cameras, and environments remains an open direction for future studies.

Despite their promise, traditional DMS face several challenges. Existing methods often rely on high-resolution eye-tracking hardware, which is expensive, complex, and difficult to scale for mass-market deployment. Specialized, standalone eye-tracking hardware can cost between $1000 and $70,000 [4,5]. These systems typically consist of complete hardware setups, often in the form of glasses, along with the associated software for measurement and analysis. However, integrating such additional hardware into a vehicle is not feasible. Furthermore, these systems are sensitive to environmental variations, such as changes in lighting, and driver-induced factors, such as head movements, limiting their effectiveness in real-world scenarios. Therefore, intensive research is being conducted to develop and evaluate low-cost eye tracking systems [6,7]. Low-cost eye tracking solutions often rely on standard webcams and open-source software. The most affordable systems are available for as little as approximately €100, although the price varies significantly depending on the desired accuracy. For instance, Fukuda et al. [8] combine image processing techniques with geometric modeling to enable robust and precise gaze estimation. Lemahieu et al. [9] utilize an infrared light source to enhance the contrast between the pupil and the rest of the eye.

Approaches that combine low-cost hardware with artificial intelligence (AI) open up additional interesting application possibilities. An approach proposed by Huang et al. [10] combines object detection based on Regions with Convolutional Neural Network (R-CNN) features to identify facial regions and initial eye positions, followed by a recurrent learning module that further refines the initial eye shapes. The method developed by Krafka et al. [11] consists of two main stages: First, facial landmarks are extracted using DeepLabCut (DLC), an open-source motion capture tool that is used here to extract relevant facial features from webcam video frames. In the second step, gaze position is estimated using a neural network (GazeNet), which processes the extracted landmarks to determine the gaze position. Recent advances in deep learning have introduced hybrid architectures for gaze estimation that leverage attention mechanisms. For instance, Li et al. proposed a model based on strip pooling and multi-criss-cross attention networks that achieves high accuracy on benchmark datasets by capturing both global and local dependencies in eye images [12]. Such neural approaches, while powerful, require extensive training data and computational resources, which makes their deployment on embedded or low-cost driver monitoring systems challenging.

This study introduces a cost-effective and computationally efficient approach to extract fixation-related eye-tracking features using standard red, green, blue (RGB) cameras, which are widely available and eliminate the need for specialized hardware. By leveraging advanced techniques such as Kalman filtering, the OPTICS algorithm, and affine transformations, the developed pipeline mitigates noise and enhances fixation analysis.

The primary objective of this study is to develop a robust and accessible method for driver state assessment in systems like DMS. The extracted fixation features are designed to provide parameters that may serve as indicators of cognitive load, attention, and emotional states. Preliminary laboratory tests in a controlled table-top setup, where participants viewed both critical and non-critical driving scenarios, already indicated that the proposed pipeline can distinguish between these conditions based on fixation dynamics. These exploratory results support the potential of the method for cognitive state estimation, but comprehensive validation with physiological and behavioral measures remains future work.

In addition to safety applications, the extracted features have the potential to optimize user experience and facilitate data-driven monetization opportunities, an emerging market expected to generate up to $400 billion annually by 2030 [3]. The following sections detail the theoretical basis of fixation analysis, the development of our feature extraction method, and experimental results that demonstrate its robustness and applicability in diverse, real-world conditions.

Unlike existing low-cost eye-tracking studies that use isolated filtering or clustering techniques, the present work integrates Kalman filtering, OPTICS clustering, and affine normalization into a single pipeline for fixation analysis. The focus is on demonstrating that this established set of techniques, when combined, provides sufficiently stable fixation estimation from standard RGB camera data without specialized hardware or infrared illumination. The study is positioned as a technical feasibility demonstration rather than a comprehensive performance evaluation.

## 2. Eye Fixation: A Window into Visual and Cognitive Processes

Fixation is a central aspect of visual perception. Its primary purpose is to stabilize a specific point in the visual field so that the image of this point remains consistently projected onto the fovea, the central region of the retina with the highest visual acuity. The human eye spends approximately 90% of its time in a state of fixation [13]. Fixation is a key parameter in eye-tracking research, offering insights into visual attention and cognitive processing. A fixation occurs when the eyes remain relatively stable and focused on an object to analyze it. Fixations represent periods during which the eyes are stationary and visual information is acquired. On average, this process occurs three to four times per second [14]. Fixation duration, ranging from 100 to 600 ms with an average of 200–400 ms [14,15,16], is significantly influenced by the availability and complexity of visual information [14]. Fixation enables the continuous and detailed acquisition of information from the observed object. Eye-tracking features offer valuable insights into cognitive load, emotional states, attention, distraction, and decision-making processes [17,18].

### 2.1. Cognitive and Perceptual Load

There is a fundamental connection between oculomotor activity and cognitive load: the higher the cognitive load, the longer the fixation duration. Oculomotor activity refers to the movement and control of the eyes, including saccades, fixations, and smooth pursuit movements. The following studies support this understanding. De Greef et al. [19] tested participants under three scenarios: one with a cognitive load well below the average, one with an average load, and one involving significant overload. For mental loads below the average, the fixation duration was on average 39 ms shorter, whereas during overload, it was 46 ms longer compared to the average fixation duration under normal cognitive load. Liu et al. [15] indicates that mental workload can be categorized into two types: perceptual load and cognitive load. Perceptual load refers to the amount of visual information processed simultaneously, such as recognizing multiple objects in a cluttered scene, whereas cognitive load involves higher-order tasks such as reasoning or memory retention. The authors support this distinction by observing opposing effects on fixation parameters for these two types. High perceptual load resulted in shorter fixation durations and a higher fixation frequency, i.e., more fixations. Conversely, high cognitive load led to longer fixation durations and a lower fixation frequency. These contrasting effects are consistent with load theory and demonstrate that fixation-related parameters can be utilized to index mental workload across different levels of processing (perceptual and cognitive) [15]. The ability to detect changes in fixation parameters is crucial for DMS, as cognitive load directly affects driver attention and safety. By leveraging the cost-effective RGB-based method proposed in this paper, fixation parameters can enable real-time cognitive load assessment, supporting adaptive interventions to enhance driver performance and prevent accidents.

### 2.2. Emotional States

The following section summarizes research findings that demonstrate the relationship between fixation parameters and emotional states. The primary insight is that longer fixation durations may indicate heightened emotional arousal (physiological and psychological activation) or stronger emotional valence (positive or negative intensity of emotion). Emotional stimuli, especially threatening ones, affect the temporal and spatial dynamics of oculomotor movements, as demonstrated by Mulckhuyse et al. [20]. Notably, covert attention cannot be detected via oculomotor movements. Covert attention refers to focusing attention on a specific location within the visual field without directing eye movements toward it. This implies that attention can be directed toward an object while the gaze remains fixed elsewhere. In contrast, overt attention can be effectively detected through oculomotor movements. Overt attention involves the deliberate alignment of eye movements and gaze toward a specific target or object in the visual field, meaning both the eyes and attention are directed at the same target. Herten et al. found in [16] that stressed participants exhibited longer fixation durations and a higher number of fixations on central objects compared to a control group. This suggests that stress induces more intensive visual exploration of relevant objects. Stress causes “attentional narrowing”, where attention focuses on salient stimuli while neglecting less relevant ones. This narrowing of attention explains the prolonged fixation on central objects perceived as more important. Emotion extraction from eye-tracking data often uses sensor fusion, combining features like pupil diameter, position, fixation duration, range of movements, and responses, which have proven especially useful. Studies of Lim et al. [21] and López-Gil et al. [22] highlight that combining eye-tracking features with electroencephalography (EEG) data yields high classification accuracy for basic emotions. Guo et al. [23] show that using only eye-tracking features, neural networks achieve classification accuracies of up to 60% for the following five emotions: happiness, sadness, fear, disgust, and neutral. These findings underscore the importance of fixation-related parameters for understanding emotional states, particularly in high-stress environments, such as driving. The proposed eye-tracking method offers a cost-effective and scalable solution for extracting these parameters. By integrating such capabilities into DMS, the system can dynamically adjust its interventions, such as issuing alerts during periods of emotional distress or adapting in-vehicle displays to minimize distractions. This approach enhances both driver safety and system adaptability without requiring expensive, high-resolution eye-tracking hardware.

### 2.3. Attention, Distraction, and Decision-Making

Eye movements provide valuable insights into drivers’ attention and behavior, particularly in hazardous situations. Velichkovsky et al. [24] analyzed fixation durations during simulated dangerous driving scenarios. Data were collected from participants who were exposed to dangerous driving scenarios in a driving simulator. The authors analyzed fixation durations around the exact moment of the hazardous event. It was observed that fixation duration at the time of the hazardous situation was significantly longer. Building on these findings, Glaholt et al. [25] examined the relationship between fixation durations and decision-making processes. In a two-alternative forced-choice (2AFC) experiment, sixteen participants were asked to select between two visual stimuli presented for either 200 or 400 ms. The results revealed that fixation durations on chosen objects were significantly longer than those on unselected objects, with this bias becoming more pronounced as exposure time increased. These findings suggest that visual attention, as reflected in fixation durations, plays a critical role in cognitive processing during decision-making tasks. The ability to detect changes in fixation durations during hazardous situations or decision-making tasks highlights the practicality of the proposed method. By capturing fixation features in real-time, this method can enhance DMS by detecting drivers’ responses during critical moments, enabling timely interventions that improve both safety and performance.

## 3. Existing Techniques for Eye-Tracking Data Analysis and Processing

The main challenge in extracting the individual eye-tracking features is to determine the pupil position as accurately as possible. Accurate detection is hindered by eye and head movements as well as landmark jitter. Landmark jitter refers to fluctuations in the detection of facial landmarks, even when the face remains stationary. Landmark jitter, also known as landmark noise, represents a critical issue in video-based facial feature detection [26]. Subtle lighting changes or minor variations in facial pose can cause the algorithm to detect slightly different landmark positions. As a result, the detected position varies slightly between frames. There are various approaches to detecting and mitigating jitter. Addressing these challenges is critical to deploying an effective DMS, where reliable fixation feature extraction is essential for real-time driver assessment.

Kalman filters are frequently used for processing and tracking eye landmarks. The study by Nugroho et al. [27] examines the effectiveness of various filtering methods (Kalman, Savitzky-Golay, and Gaussian filters) in terms of improving eye landmark detection and confirms their efficacy. The objective was to analyze the impact of these filters on the accuracy and stability of landmark detection and to evaluate their suitability for real-time applications. The results indicate that the Kalman filter achieves the lowest error rates, the highest computational efficiency, and the best jitter reduction. The paper by Bagherzadeh et al. [28] presents an algorithm for enhancing the efficiency of eye-tracking systems using a multi-model Kalman filter. While classical Kalman filters employ a single model for object tracking, the multi-model approach leverages multiple models to achieve a more accurate estimation of eye position. The algorithm integrates state models for velocity, acceleration, and rotation rate, resulting in significantly improved accuracy. Komogortsev et al. [29] developed the Attention Focus Kalman filter (AFKF), which enhances gaze control by noise suppression, eye movement detection, and attention prediction. The Kalman filter is used to reduce noise in gaze position data and to enable more accurate predictions of the attentional focus. It distinguishes between fixations, saccades, and smooth pursuits, thereby improving signal quality and perceptual compression.

The use of OPTICS has been less frequently reported in research. Johnson [30] investigated which clustering methods are best suited for accurately identifying fixations within a 3D Virtual Reality (VR) environment. For this purpose, various clustering algorithms were applied to eye-tracking data collected in a custom-developed VR environment. The following algorithms were tested: Density-Based Spatial Clustering of Applications with Noise (DBSCAN), OPTICS, Balanced Iterative Reducing and Clustering using Hierarchies (BIRCH), Affinity Propagation, and Mean Shift. DBSCAN and OPTICS achieved the best overall results, as they accurately identified the number of fixations and their cluster centers were close to the expected fixation points. The study by Naqshbandi et al. [31] investigates the automatic clustering of eye-tracking data for the classification of cognitive tasks using machine learning. Two clustering methods, k-means and OPTICS, were employed to group fixation points. The results indicate that OPTICS consistently achieves higher clustering accuracy than k-means while forming more intuitive clusters that better correspond to actual gaze trajectories. In the work of Li [32], OPTICS was examined as an alternative to DBSCAN for extracting fixations from the collected eye-tracking data. Although OPTICS enables a more precise fixation classification than DBSCAN, it was not integrated into the described eye-tracking prototype system due to its high computational complexity. DBSCAN proved to be a more practical alternative, as it operates faster while still delivering good results.

Affine transformations are primarily used in image processing, computer graphics, robotics, machine learning, geoinformation, physics, and statistics. They are also occasionally applied in eye-tracking. The affine transformation is used by Narcizo et al. [33] to map eye features into a normalized coordinate system and correct distortions. This is part of the method for compensating the camera position and contributes to reducing the non-coplanarity between the eye plane and the observed plane. The authors demonstrate that this approach enables more accurate gaze estimation by minimizing geometric distortions caused by varying camera orientations. Hu et al. [34] employ affine transformations in transfer learning to adapt the features of the pre-trained iTracker model to a new domain. The iTracker model is a CNN-based eye-tracking system that has been pre-trained on a large dataset and is adapted to new scenarios and application conditions through transfer learning. By applying affine transformations such as scaling, translation, and rotation, the pre-trained knowledge is preserved while the model adapts more effectively to new data. This approach enhances the accuracy of the eye-tracking system.

The methodology presented is a combination of three sequentially executed steps: first the Kalman filter, followed by OPTICS, and finally an affine transformation. This approach could not be found in the literature. In the following sections, both the individual steps and the overall method are described in detail.

## 4. Proposed Feature Extraction Method for Eye-Tracking Data

**Figure 1:**
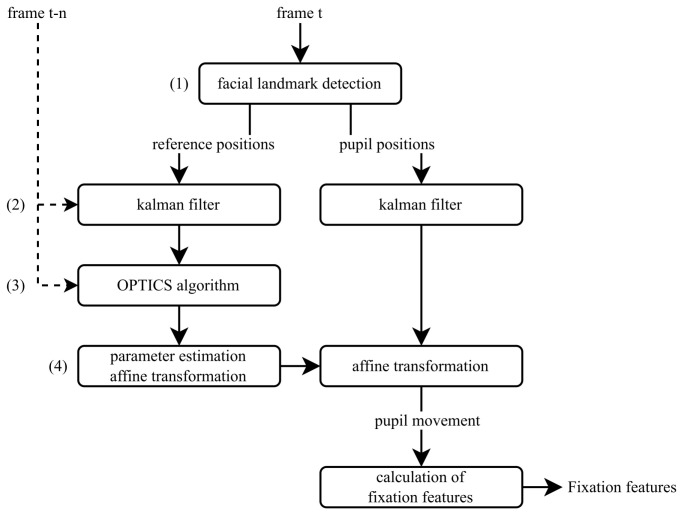
Proposed method: Calculation sequence for each video frame.(1) Facial landmark detection provides reference and pupil positions. (2) Kalman filtering is applied to stabilize landmark trajectories. (3) OPTICS clustering identifies fixation candidates within a temporal window. (4) Affine transformation parameters are estimated to normalize pupil motion before fixation feature extraction.

This section introduces a practical method for determining eye-tracking fixation parameters, which can be utilized to assess driver conditions in real time. The presented approach does not require high-resolution camera data or specialized eye-tracking hardware to capture detailed representations of the eye and pupil. Conventional eye-tracking hardware achieves this by positioning the camera sensor in close proximity to the eye, such as within a glasses frame. In contrast, the presented method operates at a significantly greater distance of 20–80 cm, resulting in substantially lower pupil resolution. A standard RGB camera operating in the visible light spectrum is used, capturing images at 30 frames per second (FPS) without the need for exceptionally high camera resolution. This paper presents an end-to-end pipeline structured as a four-part workflow, as shown in Figure 1, to address these challenges.

### 4.1. Data Preprocessing: Extraction and Noise Reduction

The prerequisite for the developed pipeline is video data in which the face of a person is visible. For each video frame, the method processes a sequence of calculations to extract fixation features, as illustrated in Figure 1.

#### 4.1.1. Facial Landmark Detection

The first step involves determining the position of facial landmarks. In principle, any face-recognition algorithm can be used for this purpose. However, algorithms with higher baseline accuracy are more likely to yield superior results. For this application, two specific conditions must be satisfied:

- The algorithm must detect the center of the pupil and return the coordinates of this position.
- The algorithm must identify the outline of the eyes or points near the outline of the eyes, with at least four points per eye, and return the coordinates of these positions.

**Figure 2:**
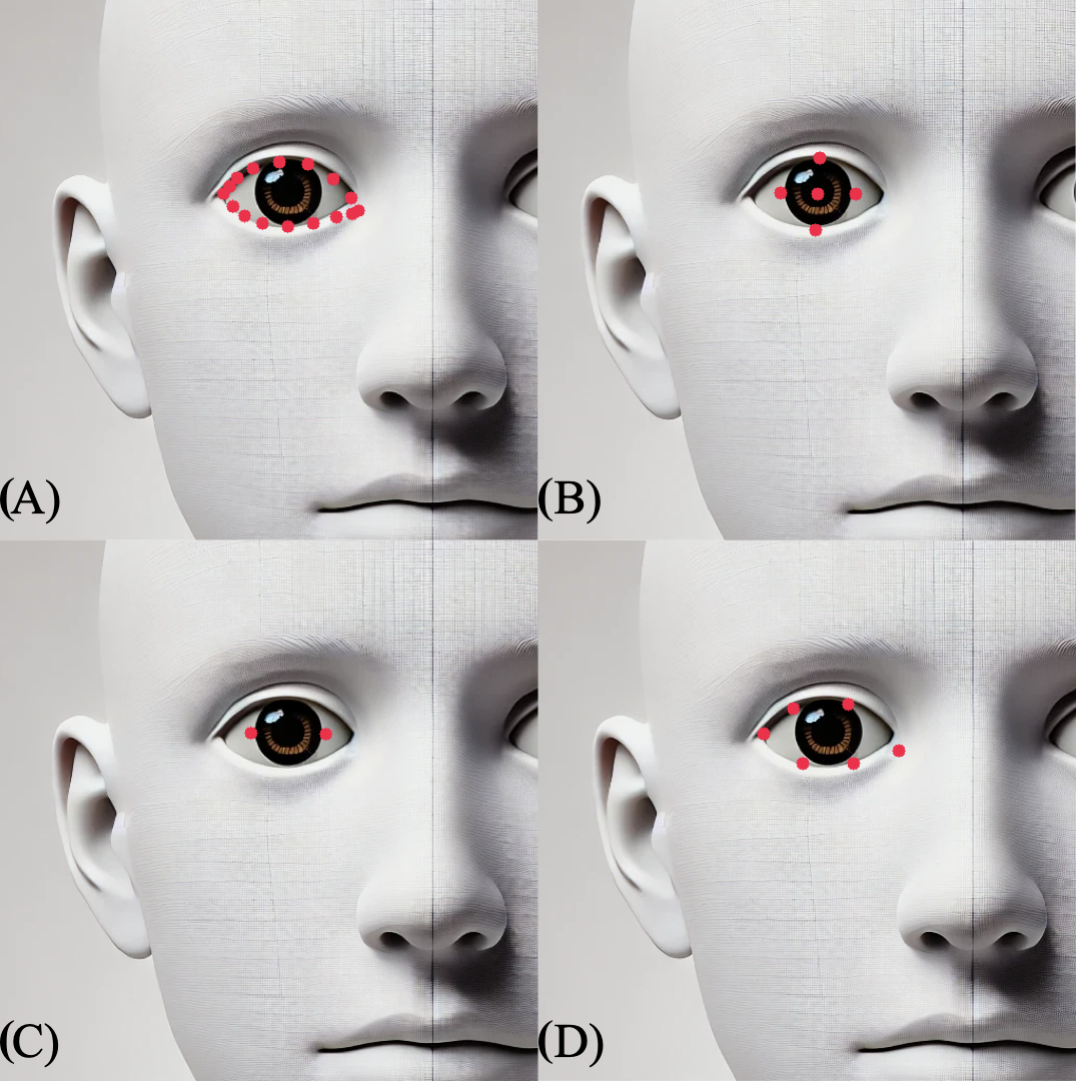
The landmark positions used by the MediaPipe algorithm are specified here for the right side of the face; the positions for the left side are derived through analogous mirroring [35]. The red dots indicate the exact landmark coordinates returned by the MediaPipe model. (**A**) Reference positions—[33, 246, 161, 160, 159, 158, 157, 173, 133, 155, 154, 153, 145, 144, 163, 7], (**B**) Pupil positions—[469, 470, 471, 472, 468], (**C**) Horizontal reference positions—[471, 469], (**D**) EAR (Eye Aspect Ratio) positions—[33, 160, 158, 133, 153, 144]. The face shown in this figure is an AI-generated illustration created using OpenAI ChatGPT (GPT-4.1) for demonstration purposes.

In this paper, the MediaPipe algorithm [35] is used. The MediaPipe algorithm was chosen because it computes a large number of facial landmark positions, particularly in the area of the eyes, without requiring additional procedures to increase the number of facial landmark positions. The landmarks used for the analysis are shown in Figure 2.

For each frame, the reference positions of the landmarks and the pupil positions are determined for both eyes. These reference positions enhance the accuracy of the pupil position calculations while minimizing noise and jitter, as demonstrated below. The position of the landmark *k* at the frame *t* is presented by the vector $P_{t,k}$, which is derived from its *x*- and *y*-coordinates. This can be expressed as (1)

(1) $$\begin{array}{c} P_{t,k}=\left[\begin{array}{c} x_{t,k}\\ y_{t,k} \end{array}\right],\;\mathrm{for}\;t\in \left\{1,2,\ldots ,T\right\}\;\mathrm{and}\;k\in \left\{1,2,\ldots ,K\right\}. \end{array}$$

where *t* is the frame index, *T* is the total number of frames, *k* is the landmark index, *K* is the total number of landmarks, and $x_{t,k}$ and $y_{t,k}$ are the respective coordinates of the landmark *k* in frame *t*.

#### 4.1.2. Kalman Filter

The raw landmark positions, including both the reference and pupil positions, are processed using a Kalman filter. The Kalman filter is a recursive algorithm that estimates the state of a dynamic system, even in the presence of significant measurement noise. It achieves this through two iterative steps: correction and prediction. The correction step updates the state vector based on measurements, while the prediction step estimates future states using system dynamics [36,37,38].

This algorithm, when applied to the coordinates of the individual positions of the landmarks, involves the below steps.

##### Initialization

All required variables and matrices are initialized. The time step dt is normalized to 1, assuming measurements are taken at equal intervals. The state transition matrix $A_{d}$ is defined as shown in Equation (2). Similarly, the observation matrix *C*, process noise covariance matrix Q(k), measurement noise covariance matrix R(k), and initial estimation covariance matrix $\hat{P}\left(k\right)$ are given in Equations (3), (4), (5) and (6), respectively.

(2) $$\begin{array}{c} A_{d}=\left(\begin{array}{cc} 1 & dt\\ 0 & 1 \end{array}\right) \end{array}$$

(3) $$\begin{array}{c} C=\left(\begin{array}{cc} 1 & 0 \end{array}\right) \end{array}$$

(4) $$\begin{array}{c} Q\left(k\right)=\left(\begin{array}{cc} 1 & 0\\ 0 & 1 \end{array}\right) \end{array}$$

(5) $$\begin{array}{c} R\left(k\right)=\left(\begin{array}{c} 100 \end{array}\right) \end{array}$$

(6) $$\begin{array}{c} \hat{P}\left(k\right)=\left(\begin{array}{cc} 1 & 0\\ 0 & 1 \end{array}\right) \end{array}$$

##### Determine Initial State

To reduce noise and provide a stable starting point, the initial position is calculated as the mean of the first three positions. The initial velocity is determined based on the difference between the first and third positions. The calculated initial state, which includes position and velocity, is expressed in Equation (7).

(7) $$\begin{array}{c} \hat{x}\left(0\right)=\left(\begin{array}{c} {\mathrm{position}}_{\mathrm{init}}\\ {\mathrm{velocity}}_{\mathrm{init}} \end{array}\right) \end{array}$$

##### Kalman Filter Loop

In the correction loop, the first step involves calculating the difference between the predicted and actual positions Δy(k), as shown in Equation (8). The Kalman gain K(k), which adjusts the weight of the predicted versus observed measurements, is computed as shown in Equation (9). In the predictions loop, the new state x(k+1) is predicted using Equation (10), and the covariance matrix P(k+1) is updated as Equation (11). The result is a Kalman-filtered position $P_{(K),t}$ for each frame *k* and each landmark *i*, represented as Equation (12) [37,38].

(8) $$\begin{array}{c} \Delta y\left(k\right)=y\left(k\right)-C\cdot \hat{x}\left(k\right) \end{array}$$

(9) $$\begin{array}{c} K\left(k\right)=\frac{P\left(k\right)\cdot C^{T}}{C\cdot P\left(k\right)\cdot C^{T}+R\left(k\right)} \end{array}$$

(10) $$\begin{array}{c} x\left(k+1\right)=A_{d}\cdot x\left(k\right) \end{array}$$

(11) $$\begin{array}{c} P\left(k+1\right)=A_{d}\cdot P\left(k\right)\cdot A_{d}^{T}+Q \end{array}$$

(12) $$\begin{array}{c} P_{(K),t}=\left(x_{i,k},y_{i,k}\right) \end{array}$$

#### 4.1.3. OPTICS Algorithm

An OPTICS algorithm is a density-based method for cluster analysis. Cluster analysis aims to identify groups of data points (clusters) in which members are as similar as possible to each other while being as dissimilar as possible to points in other clusters. The OPTICS approach extends density-based clustering algorithms, such as DBSCAN, to identify clusters with significantly varying densities. This is also the reason why OPTICS is used instead of DBSCAN, as the fixation centers can have varying cluster densities. Moreover, it eliminates the need to predefine the number of clusters. The core idea of the algorithm is to visit all points in the dataset sequentially to compute the core distance and reachability distance for each point. The core distance is fundamental to determining the density within a point’s neighborhood and influences the algorithm’s clustering decisions.

The OPTICS algorithm has been specifically adapted to enhance the detection and identification of fixations. Rather than processing all coordinates from the entire dataset, a sliding window centered on the position at frame *t* is utilized. Consequently, the dataset passed to the OPTICS algorithm for each frame is defined as $\left\{P(K),t-n/2,\ldots ,P_{(K),t+n/2}\right\}$ where *n* represents the number of frames in the window. The value of *n* is determined experimentally based on the FPS. Current findings indicate that time spans ranging from 0.5 to 4 s provide the best results. The minimum number of neighboring points MinPts is also determined experimentally as: MinPts=fps·0.3. For instance, at 30 FPS, at least 10 points are required to classify a point as a core point. To identify clusters based on the computed reachability distances, at least 25% of the dataset is required to belong to a cluster for recognition. For instance, at 30 FPS and a 3 s sliding window, this corresponds to at least 23 frames. After executing the OPTICS algorithm, a new position is recalculated for each frame as follows Equation (13).

(13) $$\begin{array}{c} P_{(K,O),t}=\left\{\begin{array}{cc} \mathrm{cluster}\;\mathrm{center} & \mathrm{IF}\;\mathrm{OPTICS}\;\mathrm{cluster}\;=\;\mathrm{true}\\ \mathrm{original}\;\mathrm{position} & \mathrm{else} \end{array}\right. \end{array}$$

All experimentally determined parameters were obtained through exploratory data analysis, in which the time intervals were varied across different datasets from various samples.

#### 4.1.4. Affine Transformation

Following the clustering of reference coordinates using the OPTICS algorithm, the parameters for an affine transformation are computed, as illustrated in Figure 1. The goal of the transformation is to compensate for motion and scale variations and to standardize the representation of pupil position. The parameters for rotation, scaling, and translation are derived from the reference positions, denoted as $P_{(K,O),t}$.

##### Translation

Translation is used to align the reference positions relative to a common origin, simplifying further analysis. To determine the translation, the centroid *c* of the reference positions is computed using Equation (14):

(14) $$\begin{array}{c} c=\frac{1}{N}\sum_{i=1}^{N}P_{(K,O),i} \end{array}$$

Here, *N* denotes the number of reference points around the eye, and *i* represents the index of the positions. Consequently, the translated pupil position is given by Equation (15):

(15) $$\begin{array}{c} P_{\mathrm{pupil}}^{*}=P_{(K,O),\mathrm{pupil}}-c \end{array}$$

##### Rotation

To achieve horizontal alignment of the eye, two landmark positions are used, as illustrated in Figure 2C. The angle θ is derived from these two horizontal reference points $P_{h,\mathrm{ref},1}$ and $P_{h,\mathrm{ref},2}$ using Equation (16). The rotation matrix R is subsequently defined as shown in Equation (17).

(16) $$\begin{array}{c} \theta =\arctan\left(\frac{\Delta y_{h,\mathrm{ref}}}{\Delta x_{h,\mathrm{ref}}}\right) \end{array}$$

(17) $$\begin{array}{c} R=\left[\begin{array}{cc} \cos(\theta ) & -\sin(\theta )\\ \sin(\theta ) & \cos(\theta ) \end{array}\right] \end{array}$$

Thus, the rotated pupil position is calculated as Equation (18).

(18) $$\begin{array}{c} P_{\mathrm{pupil}}^{**}=R\cdot P_{\mathrm{pupil}}^{*} \end{array}$$

##### Scaling

To normalize the reference positions, and consequently the pupil position, the reference eye is scaled to a predefined standard width and height. The maximum extents in the x- and y-directions are computed as in Equations (19) and (20).

(19) $$\begin{array}{c} x_{\mathrm{range}}=\max\left(x_{(K,O),i}^{**}\right)-\min\left(x_{(K,O),i}^{**}\right) \end{array}$$

(20) $$\begin{array}{c} y_{\mathrm{range}}=\max\left(y_{(K,O),i}^{**}\right)-\min\left(y_{(K,O),i}^{**}\right) \end{array}$$

(21) $$\begin{array}{c} s=\min\left(\frac{1}{x_{\mathrm{range}}},\frac{1}{y_{\mathrm{range}}}\right) \end{array}$$

Equations (19) and (20) compute the spans in the x- and y-directions across all reference positions *i*. The final scaling factor, *s*, as defined in Equation (21), is chosen as the smaller of the two values to preserve the original proportions. The scaling step transforms the pupil position as Equation (22). After scaling, a final translation is applied to center the reference positions within the target area. The final standardized reference position of the pupil is then determined as in Equation (23).

(22) $$\begin{array}{c} P_{\mathrm{pupil}}^{***}=s\cdot P_{\mathrm{pupil}}^{**} \end{array}$$

(23) $$\begin{array}{c} P_{\mathrm{pupil}}^{****}=P_{\mathrm{pupil}}^{***}+\left[\frac{1}{2},\frac{1}{2}\right] \end{array}$$

### 4.2. Application of the Proposed Method

This section demonstrates the application of the proposed pipeline through specific examples. The video data was collected using a full-HD webcam from an Apple MacBook Pro 13″ (M2, 2022) [39]. Data processing and visualization were conducted with Python 3.11.8 [40].

**Figure 3:**
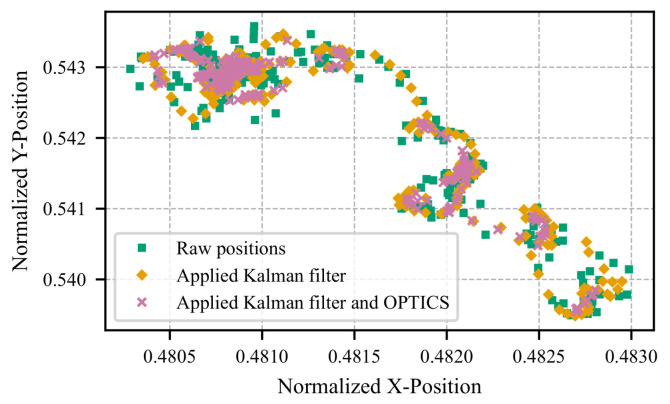
Visualization of the proposed method’s application, showing pupil coordinates for a stationary gaze directed at the screen center, without head or screen movement. Initial pupil positions from facial landmark detection are shown in green, Kalman filter-processed positions in orange, and those refined using the OPTICS algorithm in magenta. A 7.2 s segment (216 frames) is depicted.

Figure 3 illustrates the simplest test case, where the participant fixates on a stationary image displayed on the screen while minimizing head and screen movement. The computational steps are depicted, beginning with the raw pupil positions detected via facial landmark detection, followed by denoising using the Kalman filter, and finally, processing with the OPTICS algorithm. However, a noticeable drift in position remains, despite the participant maintaining a stationary gaze. This drift is primarily due to the high sensitivity of pupil detection within the small analyzed region. Small deviations in the facial landmark detection algorithm can cause significant jumps and drifts in the gaze position. Subtle factors, such as blinking (not excluded from this dataset) or slight variations in lighting, can also influence the detected position, as shown in the figure.

**Figure 4:**
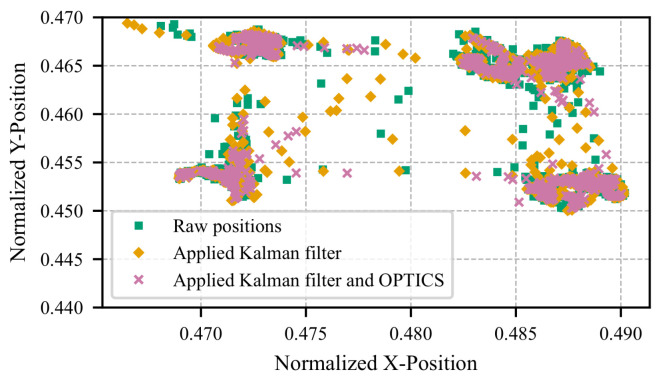
Visualization of pupil coordinates showing gaze shifts between the screen’s corners with minimal head movement. Initial pupil positions obtained via facial landmark detection are marked in green, Kalman filter-refined positions in orange, and further refined positions using the OPTICS algorithm in magenta. The visualization represents an 800-frame segment (26.7 s).

Figure 4 illustrates another example, where the participant’s gaze shifts between the corners of the screen. The effects of the applied algorithms are more pronounced in this case. Raw pupil positions from facial landmark detection exhibit noticeable jumps and fluctuations, both within and between corners. The Kalman filter significantly reduces noise, while the OPTICS algorithm further refines the central position, or “center of mass,” of each fixation. In this example, the participant circled the corners in a clockwise direction. Notably, the consistency of the fixated corner positions varies slightly between iterations. This variation is primarily due to landmark jitter and minor head movements. The affine transformation introduced in this approach has not been applied in this case, which may mitigate these effects.

**Figure 5:**
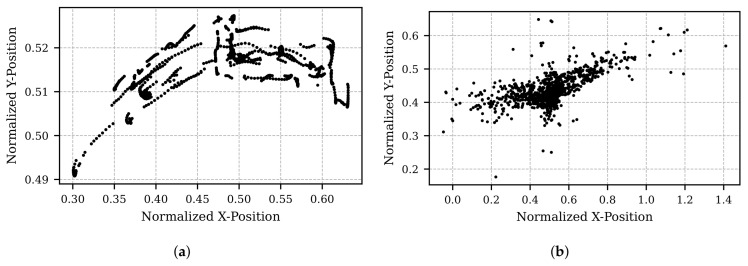
Visualization of pupil position as the subject fixates on a stationary point while the camera moves. (**a**) Pupil position after processing raw data with a Kalman filter for motion smoothing and the OPTICS clustering algorithm for artifact removal. The curved coordinates result from the camera’s offset position above the focal point. (**b**) Transformed pupil position, derived from the data in (**a**), using the affine transformation.

**Figure 6:**
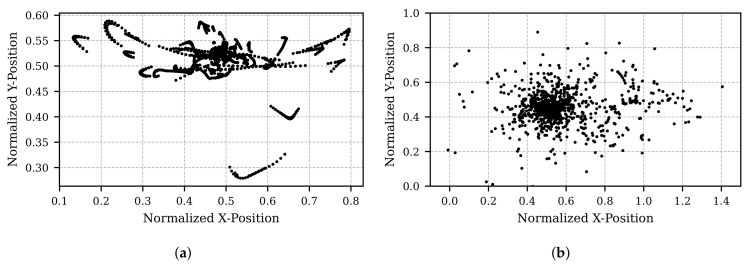
Visualization of pupil position as the subject fixates on a stationary point while deliberately moving their head in all directions. (**a**) Pupil position after processing raw data with a Kalman filter for motion smoothing and the OPTICS clustering algorithm for artifact removal. (**b**) Transformed pupil position, derived from the data in (**a**), using the affine transformation.

Figure 5 and Figure 6 illustrate the final step of the developed pipeline: the application of an affine transformation. The test data in Figure 5 represents a scenario where the screen and integrated camera are rotated, while the participant maintains a fixed gaze at a specific position with their head stationary. In contrast, Figure 6 depicts a scenario where the camera remains stationary, and the participant moves their head in various directions while continuing to fixate on a single position.

In both figures, subfigure (a) shows the pupil position after processing with the Kalman filter and OPTICS, and subfigure (b) presents the results following the affine transformation. Ideally, the transformed results in subfigure (b) should indicate a stationary gaze point in both cases. Although this is approximately achieved, residual noise persists. Nevertheless, the improvement due to the affine transformation is clearly observable when comparing subfigure (b) to subfigure (a).

### 4.3. A Method for Approximating Fixation Feature Calculations

**Figure 7:**
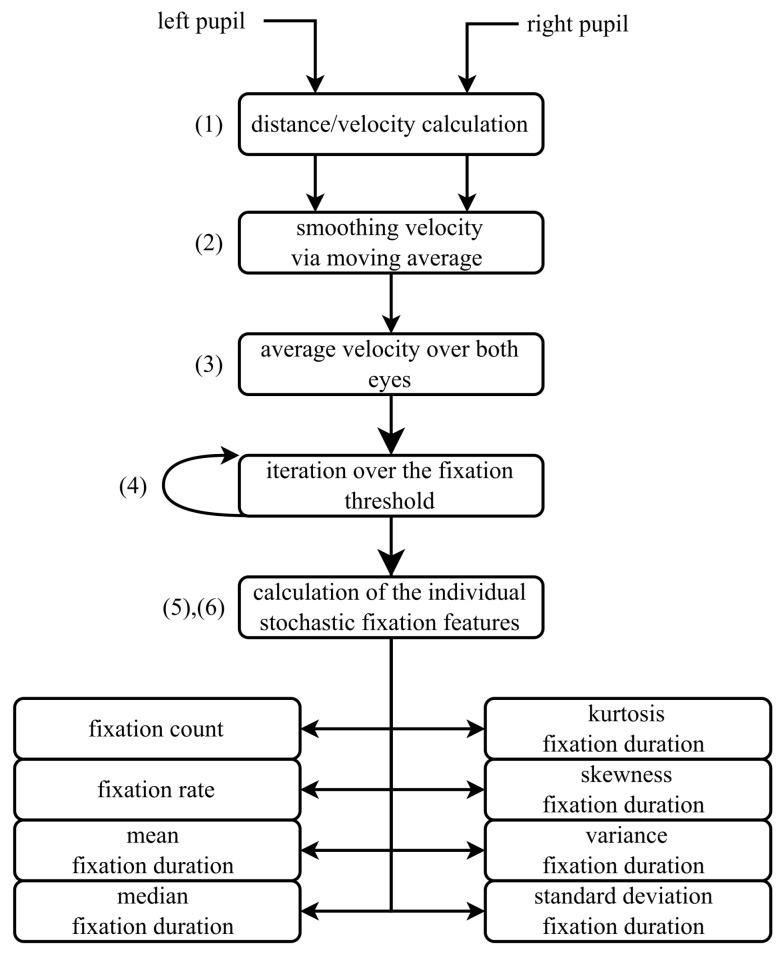
Flowchart illustrating the method for approximating fixation features. (1) Velocity and displacement of each pupil are computed. (2) The velocity signal is smoothed using a moving average. (3) The average velocity over both eyes is obtained. (4) The fixation threshold is iteratively applied to segment fixation intervals. (5) Stochastic fixation features are computed for each fixation segment. (6) The resulting features include fixation count, fixation rate, mean and median fixation duration, and statistical descriptors such as variance, standard deviation, skewness, and kurtosis.

Following data preparation during preprocessing (Figure 1), fixation-related measures are calculated as outlined in Figure 7. The preprocessing results in denoised and artifact-free coordinates for the two pupils $P_{\mathrm{pupil},\mathrm{left}}$ and $P_{\mathrm{pupil},\mathrm{right}}$.

1. The distance, and consequently the velocity, of pupil positions between consecutive frames is calculated using the Euclidean distance Formula (24).
  (24) $$\begin{array}{c} d=\sqrt{{\left(x_{t+1}-x_{t}\right)}^{2}+{\left(y_{t+1}-y_{t}\right)}^{2}} \end{array}$$
2. The velocity is then smoothed using a moving average Equation (25) [41]. The number of frames *q*, considered before and after the current frame, is determined empirically as $q=\lceil \frac{\mathrm{fps}}{30}\rceil$.
  (25) $$\begin{array}{c} \hat{d}=\frac{1}{2q+1}\left(y_{t-q}+\ldots +y_{t}+\ldots +y_{t+q}\right) \end{array}$$
3. The average velocity is calculated using the velocities of the left and right pupil positions.
4. An iterative process is used to approximate the fixation threshold, which represents the maximum pupil movement speed at which a fixation state can still be assumed. Details of this approach and its limitations are provided in Section 4.4. The threshold is iteratively adjusted until it satisfies the specified requirements for the defined time interval.
5. A boolean vector is calculated based on the fixation threshold, marking instances where the velocity exceeds the defined value.
6. In the final step, fixation features are extracted from the boolean vector of fixations. To align with literature practices, the analysis is conducted on data aggregated into 3 s bins [42]. Table 1 summarizes the calculated features and their descriptions.

### 4.4. Iterative Fixation Threshold Estimation: Necessity and Implications

Defining a fixation state requires establishing a threshold below which pupil movements are negligible and interpreted as fixation. However, complete immobility cannot be assumed due to noise and physiological factors. Absolute fixation is prevented by physiological mechanisms that induce constant minimal pupil movement, stabilizing visual perception and maintaining retinal stimuli. One key mechanism is the Troxler effect, in which stationary objects in the peripheral visual field “disappear” or appear blurred when the eyes remain fixed on a single point for an extended period. This phenomenon occurs due to sensory adaptation, where the visual system ignores stationary stimuli in the absence of new information [43,44].

Counteracting this are microsaccades, small, jerky movements of the eyes. These irregular, low-amplitude movements prevent the adaptation of photoreceptors in the retina [43,44]. Additionally, physiological nystagmus, characterized by rhythmic eye movements with rapid (saccadic) and slow phases, further prevents sensory adaptation. Often described as “tremor-like eye movements,” nystagmus displaces retinal images slightly, continuously stimulating new photoreceptors and enabling sustained visual perception [43,44].

Defining a fixation state requires establishing a threshold below which eye movements are classified as fixation. Since the magnitude of eye movement varies significantly depending on recording conditions, the fixation threshold must balance general applicability with individual adjustment to suit specific contexts.

Two parameters must be defined: the duration during which eye movement must remain below the threshold, and the numerical value of the threshold itself. Fixation durations in the literature range from 100 to 600 ms, with an average of 200–400 ms [14,15,16]. At a temporal resolution of 30 FPS, fixation must persist for at least 8 frames, corresponding to a duration of approximately 233–300 ms.

Determining the fixation threshold is inherently complex. The literature suggests that the ratio of fixation to non-fixation is approximately 90% fixation over sufficiently long periods [14,42,45,46,47,48]. The algorithm, from the method presented here, iteratively adjusts the threshold to ensure that at least 90% of the total duration is classified as fixation, with each fixation lasting a minimum of 8 frames. A tolerance of 0.25% is allowed for this criterion. If the algorithm does not converge within ${\mathrm{iter}}_{\max}$ iterations, the process is terminated. As a result, the method provides primarily quantitative outputs, with limited applicability for qualitative analysis.

**Figure 8:**
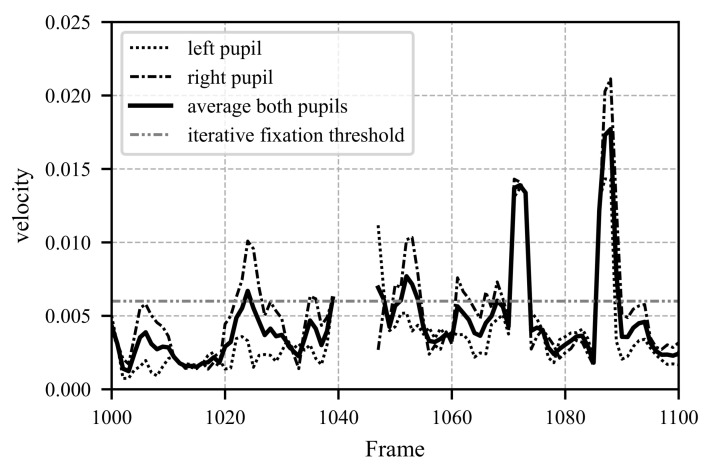
Representation of the actual pupil movement data. The presented data depict the state before and after Step 3, as illustrated in Figure 7. The shown excerpt covers 100 frames (equivalent to 3.3 s) at a frame rate of 30 FPS. Shortly after frame 1040, data on pupil movement is missing due to a blinking event, which is also visualized in Figure 9. The iteratively determined fixation threshold defines the boundary below which a fixation is assumed, provided it occurs over a minimum of 8 consecutive frames. The results indicating whether a fixation is present are shown in Figure 10.

**Figure 9:**
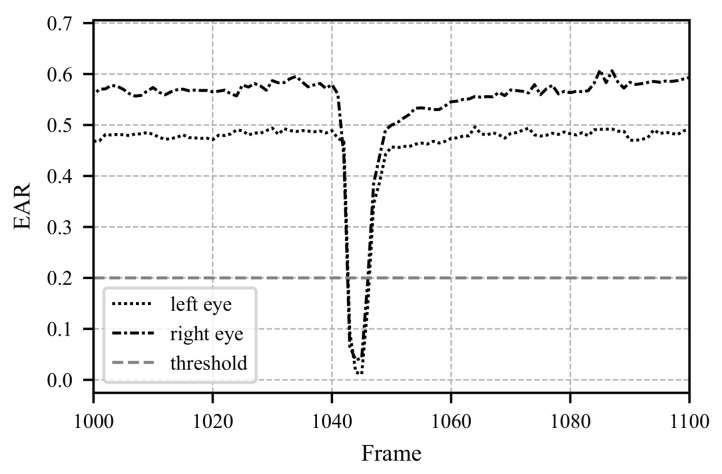
The figure shows the actual Eye Aspect Ratio (EAR) data for the same time period as in Figure 8. The EAR is calculated according to Equation (26). Starting from frame 1040, the EAR drops significantly below the threshold of 0.2, indicating that the data from this period cannot be used and that no fixation is present. In Figure 8, the data for this period is missing, and it can be observed in Figure 10 that no fixation occurs during this time.

**Figure 10:**
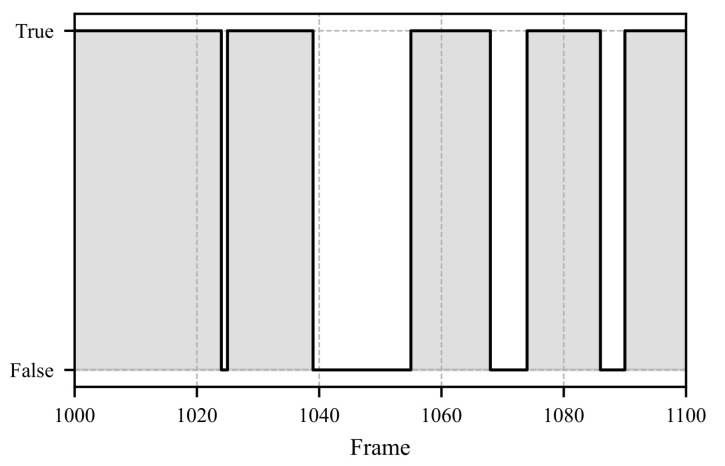
The figure shows the actual data for each frame and indicates whether a fixation is present (TRUE) or not (FALSE). The depicted time period corresponds to the one shown in Figure 8 and Figure 9. A fixation is defined when pupil movement remains below the fixation-threshold and persists for at least 8 frames, as illustrated in Figure 8.

Figure 8, Figure 9 and Figure 10 illustrate a segment spanning 100 frames (3.3 s). In Figure 8, the pupil movements of both eyes and their average are depicted. The iterative fixation threshold is shown as a horizontal gray line. When the average pupil movement remains below this threshold for more than 8 frames, the fixation is marked as “TRUE”, as seen in Figure 10. To avoid errors caused by closed eyes, the Eye Aspect Ratio (EAR) is calculated in parallel. If the EAR falls below a certain limit (e.g., during a blink), the corresponding data points related to pupil movement are excluded from further analysis. The EAR is computed as described in Equation (26).

(26) $$\begin{array}{c} \mathrm{EAR}=\frac{∥P_{2}-P_{6}∥\;+\;∥P_{3}-P_{5}∥}{2\;\cdot \;∥P_{1}-P_{4}∥}. \end{array}$$

In Equation (26) $∥P_{2}-P_{6}∥$ represents the Euclidean distance between specific points. The positions of points $P_{1}$ to $P_{6}$ are chosen as defined in the literature [49]. In the literature, the EAR, threshold is commonly set at 0.2 [49,50]. Figure 9 shows the EAR over time. Around frame 1044, a blink occurs, and the corresponding pupil movement values are discarded.

## 5. Example Results from Proposed Method

**Figure 11:**
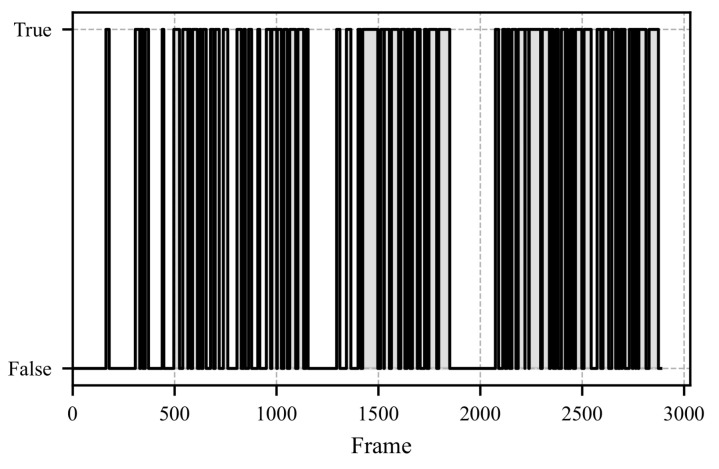
The figure illustrates the boolean fixation vector throughout the study, which spans 96.2 s. A status of “TRUE” denotes the presence of a fixation, whereas “FALSE” indicates its absence.

**Figure 12:**
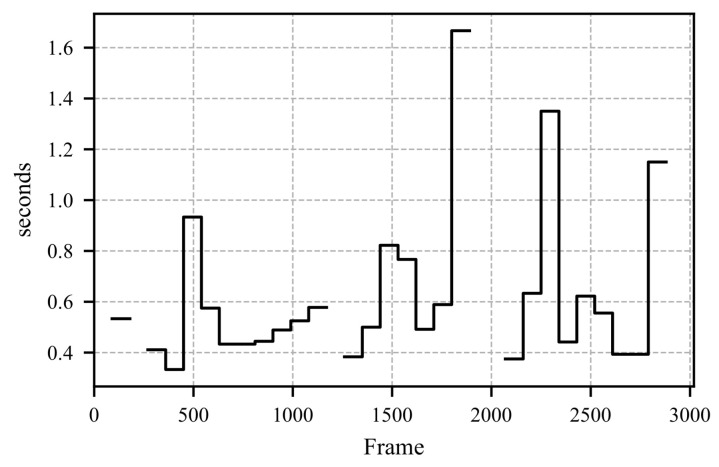
The subject observes a critical driving scenario presented on a laptop. The collected data has a duration of 96.2 s. The feature under investigation, the mean AFD, is calculated from the fixation vector (see Figure 11). Missing values occur when no fixation is detected within the calculation window, which has a duration of 3 s.

**Figure 13:**
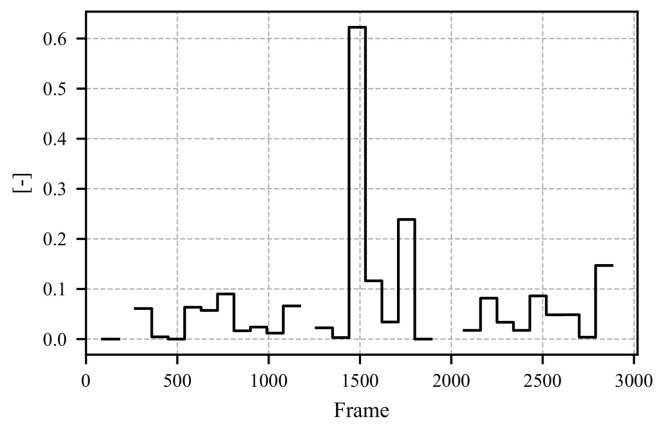
The variance of the AFD is derived from the fixation vector, shown in Figure 11. Missing values arise when no fixation is recorded within the 3 s calculation window.

**Figure A1:**
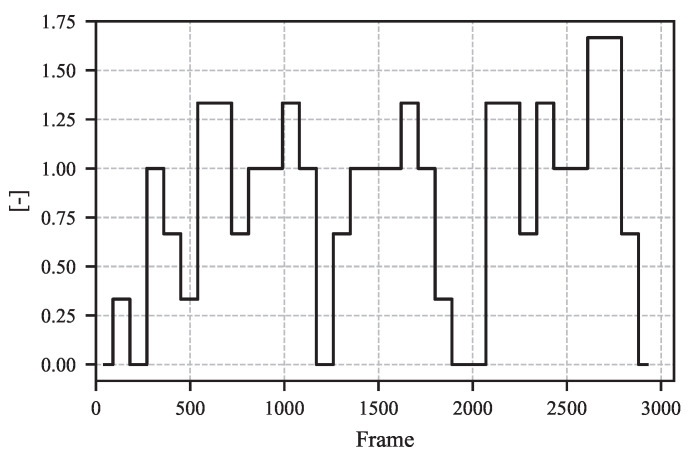
The figure illustrates the fixation rate (FR) throughout the study, which spans 96.2 s. The FR is derived from the fixation vector, shown in Figure 11.

**Figure A2:**
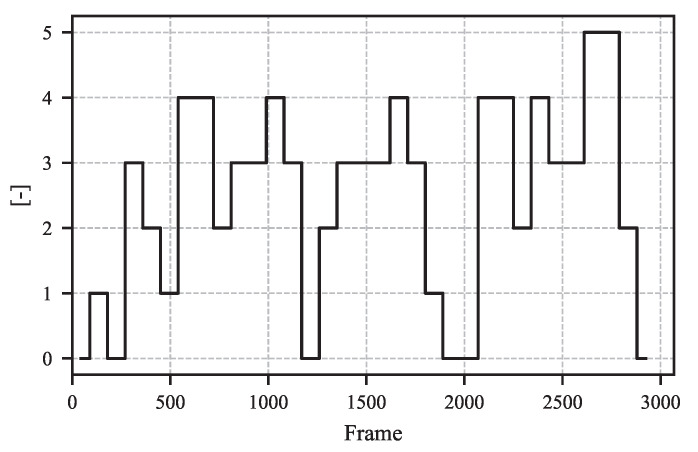
The figure illustrates the fixation count (FC) throughout the study, which spans 96.2 s. The FC is derived from the fixation vector, shown in Figure 11.

**Figure A3:**
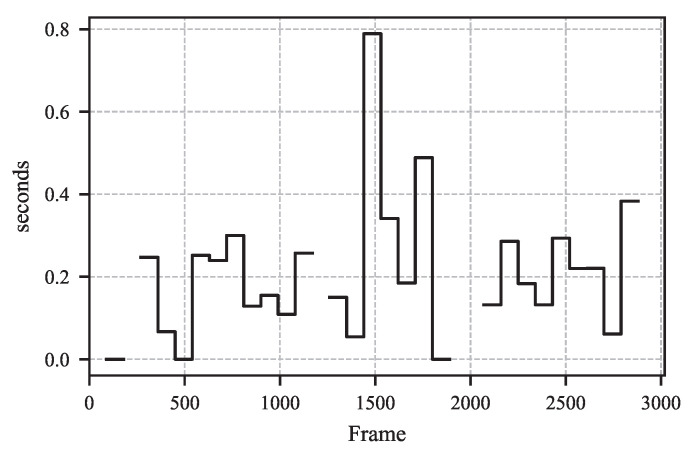
The figure illustrates the standard deviation of the AFD throughout the study, which spans 96.2 s. The standard deviation is derived from the fixation vector, shown in Figure 11. Missing values arise when no fixation is recorded within the 3 s calculation window.

**Figure A4:**
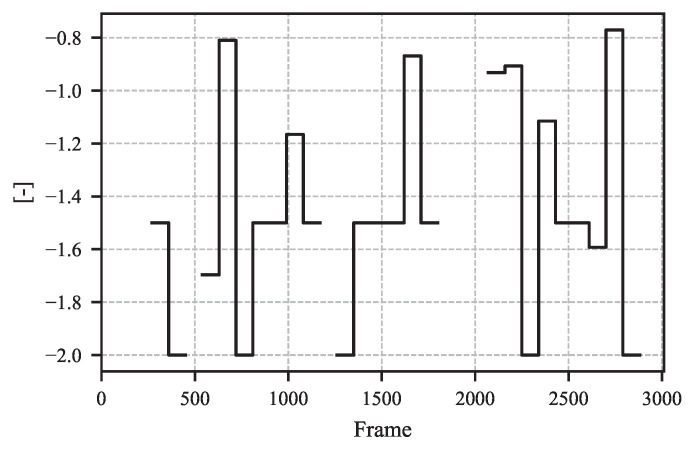
The figure illustrates the kurtosis of the AFD throughout the study, which spans 96.2 s. The kurtosis is derived from the fixation vector, shown in Figure 11. Missing values arise when no fixation is recorded within the 3 s calculation window.

**Figure A5:**
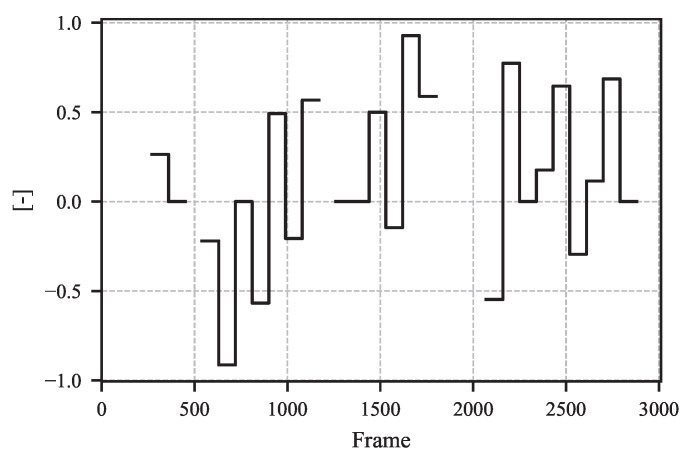
The figure illustrates the skewness of the AFD throughout the study, which spans 96.2 s. The skewness is derived from the fixation vector, shown in Figure 11. Missing values arise when no fixation is recorded within the 3 s calculation window.

To evaluate the results of the proposed method, a study was conducted in which participants were shown a video of a simulated critical driving scenario on a laptop. The features were calculated based on the fixation vector depicted in Figure 11. Figure 12 illustrates the Mean Average Fixation Duration (AFD), while Figure 13 shows the Variance of AFD. The remaining calculated features are provided in Appendix A as Figure A1, Figure A2, Figure A3, Figure A4 and Figure A5.

The video presented in the study depicts the critical scenario between frames 1500 and 2000. This scenario involves an accident scene followed by a full braking maneuver to avoid a rear-end collision. The response of the mean AFD to this scenario is shown in Figure 12. Upon the appearance of the accident scene, the participants’ mean AFD increases significantly. By the time the accident scene is reached, no further fixations can be measured (see Figure 11), making it impossible to calculate a mean AFD. A similar pattern is observed for the variance of AFD, which is shown in Figure 13; upon recognizing the critical scenario, the variance increases significantly, indicating moments with prolonged fixation durations.

## 6. Discussion

The proposed method provides a direct approach to extracting fixation-related features from video data. The present study serves as a technical feasibility demonstration rather than a large-scale evaluation. The experiments were intentionally limited to controlled conditions to isolate and assess the stability improvements gained by the integrated pipeline. Consequently, the results should be interpreted as a proof of operability under specific test scenarios. A systematic validation across subjects, lighting conditions, camera types, and real driving environments remains part of future work. Under realistic driving conditions, fluctuating illumination, head motion, and vehicle dynamics may influence the method’s robustness, which will be addressed in subsequent studies. While the this method focuses on fixation-based indicators of attention and cognitive state, it does not yet address extreme physiological or emotional conditions such as drowsiness, fainting, or crying. These states involve additional cues such as eyelid closure dynamics, facial muscle activation, and tear-induced reflections, which are outside the current scope but could be incorporated in future multimodal extensions that combine fixation data with eyelid and facial feature analysis. For real-world applications, the use of near-infrared cameras instead of conventional visible-spectrum cameras is recommended to reduce the influence of lighting conditions. In future implementations, adaptive calibration and automatic compensation for head pose variations may further enhance robustness and stability across diverse environments.

The presented method for extracting fixation features enables the identification of relationships between fixation characteristics, cognitive load, and emotional states. In a preliminary laboratory evaluation conducted outside the main study, participants observed critical and non-critical driving scenarios while fixation behavior was recorded using the proposed pipeline. The extracted features showed distinct differences between the two conditions, indicating sensitivity to variations in cognitive load. These results are promising yet exploratory, as they were obtained from a limited sample under controlled conditions. They therefore serve as indicative evidence of the method’s potential rather than as a validated confirmation of cognitive state estimation. The present evaluation is descriptive and does not include statistical analysis due to the exploratory scope and limited sample size. Future large-scale studies will incorporate statistical methods to quantify the observed effects and validate the relationships between fixation parameters and cognitive state indicators.

This information can be useful for optimizing DMS. When observing the driver with a camera, the processing can be entirely software-based, without requiring specialized camera hardware. A key advantage of this method is that it imposes no special requirements on the camera data, allowing for a simple and scalable implementation. If the necessary hardware is already installed in the vehicle, the computation can be fully integrated as a software update. It is important to note that the extracted features do not achieve the quality possible with specific and higher-quality camera data. The aim of the method is to extract as much information as possible under the given conditions.

Although the present study focused on qualitative evaluation and technical feasibility, a quantitative benchmarking of the pipeline will be an important next step. Future work will therefore include systematic comparisons with other low-cost eye-tracking approaches to assess relative accuracy, computational efficiency, and robustness under varying conditions. Such analyses will allow a clearer positioning of the developed approach within the broader context of existing driver monitoring solutions.

The following limitations of the method should be noted. First, the method lacks strict real-time capability because various features are calculated based on time intervals, and parts of the computation rely on time-based data. Strict real-time capability is therefore not yet achieved, although this may not be necessary for many applications. The efficiency of the computation can still be improved. For example, the calculation of the parameters for the affine transformation could be initiated only when necessary, that is, for new frames showing significant changes or motion. A further limitation of the method is its reliance on calibration for the specific driver and environment. The method is dependent on specific parameters, such as the distance between the camera and the driver. A potential solution could involve conducting a baseline measurement at the start of a trip as a reference for subsequent trips. Additionally, saving a “driver profile” across multiple trips may be a practical approach.

## 7. Conclusions

This paper presented a computationally efficient proof of concept for extracting fixation-related features using standard RGB cameras and widely accessible algorithms. The study demonstrated the technical feasibility of stabilizing fixation estimation with low-cost hardware under controlled conditions. The evaluation was intentionally limited in scope and designed to isolate the effects of Kalman filtering, OPTICS clustering, and affine normalization on gaze stability.

The results indicate that reliable fixation estimation can be achieved with conventional RGB cameras, providing a foundation for future low-cost driver monitoring solutions. However, the findings cannot be generalized beyond the tested setup. Broader validation across subjects, lighting conditions, and camera configurations is required to confirm robustness and reproducibility.

Future work will focus on large-scale experiments under real driving conditions, the integration of near-infrared cameras to mitigate lighting influences, and adaptive calibration strategies to compensate for driver and environmental variability. The proposed pipeline therefore serves as a basis for continued research toward fully validated, scalable, and software-based driver monitoring systems. Moreover, the presented proof of concept was implemented and evaluated in a desktop environment. Future work will therefore include the deployment of the algorithm on embedded hardware architectures to assess real-time performance and computational feasibility within in-vehicle systems. Initial exploratory observations from a separate laboratory setup further suggested that the proposed method can differentiate between critical and non-critical driving scenes based on fixation dynamics. This supports the potential applicability of the approach for future cognitive load estimation studies once formal validation with larger participant groups and multimodal data is performed.

## Figures and Tables

**Table 1:** Description of Calculated Features Related to Fixation Durations.

Feature Name	Description
Fixation Count (FC)	Total number of fixations during the observation period.
Fixation Ratio (FR)	Proportion of fixation time relative to the total observation duration.
Mean Average Fixation Duration	Average length of individual fixations.
Median Average Fixation Duration	Representing the middle value when all durations are ordered.
Standard Deviation of Average Fixation Duration	Measure of variability in fixation durations.
Variance of Average Fixation Duration	Measure of the spread of fixation durations.
Skewness of Average Fixation Duration	A statistical metric assessing the asymmetry of the distribution of fixation durations.
Kurtosis of Average Fixation Duration	A statistical metric assessing the distribution’s peakedness or tail-heaviness.

## Data Availability

This article includes the original contributions of this research. If you have any questions, please contact the corresponding author.

## Abbreviations

The following abbreviations are used in this manuscript:

2AFC	Two-Alternative Forced-Choice
ADAS	Advanced Driver Assistance Systems
AFD	Average Fixation Duration
AFKF	Attention Focus Kalman Filter
AI	Artificial Intelligence
BIRCH	Balanced Iterative Reducing and Clustering using Hierarchies
DBSCAN	Density-Based Spatial Clustering of Applications with Noise
DMS	Driver Monitoring System
EAR	Eye Aspect Ratio
EEG	Electroencephalography
EU	European Union
FPS	Frames Per Second
HVAC	Heating, Ventilation, and Air Conditioning
NIR	Near-Infrared
OPTICS	Ordering Points To Identify the Clustering Structure
R-CNN	Regions with Convolutional Neural Networks
RGB	Red, Green, Blue
VR	Virtual Reality
